# CDKN2A promoter methylation enhances self-renewal of glioblastoma stem cells and confers resistance to carmustine

**DOI:** 10.1007/s11033-024-09247-5

**Published:** 2024-03-05

**Authors:** Jing Wang, Yan-feng Xi, Qi Zhao, Jiang-hong Guo, Zhen Zhang, Mao-bai Zhang, Jiang Chang, Yue-qin Wu, Wen Su

**Affiliations:** 1 Department of Pathology, Cancer Hospital Affiliated to Shanxi Medical University/Shanxi Province Cancer Hospital/ Shanxi Hospital Affiliated to Cancer Hospital Chinese Academy of Medical Sciences, Taiyuan, 030013 Shanxi China; 2 Department of Neurosurgery, Cancer Hospital Affiliated to Shanxi Medical University/Shanxi Province Cancer Hospital/ Shanxi Hospital Affiliated to Cancer Hospital Chinese Academy of Medical Sciences, Taiyuan, 030013 Shanxi China; 3 Department of Medical Laboratory, Cancer Hospital Affiliated to Shanxi Medical University/Shanxi Province Cancer Hospital/ Shanxi Hospital Affiliated to Cancer Hospital Chinese Academy of Medical Sciences, Taiyuan, 030013 Shanxi China

**Keywords:** CDKN2A, Glioblastoma, Gene expression, Cell viability, Promoter methylation

## Abstract

**Background:**

Glioblastoma, a highly aggressive form of brain cancer, poses significant challenges due to its resistance to therapy and high recurrence rates. This study aimed to investigate the expression and functional implications of CDKN2A, a key tumor suppressor gene, in glioblastoma cells, building upon the existing background of knowledge in this field.

**Method:**

Quantitative reverse transcription PCR (qRT-PCR) analysis was performed to evaluate CDKN2A expression in U87 glioblastoma cells compared to normal human astrocytes (NHA). CDKN2A expression levels were manipulated using small interfering RNA (siRNA) and CDKN2A overexpression vector. Cell viability assays and carmustine sensitivity tests were conducted to assess the impact of CDKN2A modulation on glioblastoma cell viability and drug response. Sphere formation assays and western blot analysis were performed to investigate the role of CDKN2A in glioblastoma stem cell (GSC) self-renewal and pluripotency marker expression. Additionally, methylation-specific PCR (MSP) assays and demethylation treatment were employed to elucidate the mechanism of CDKN2A downregulation in U87 cells.

**Result:**

CDKN2A expression was significantly reduced in glioblastoma cells compared to NHA. CDKN2A overexpression resulted in decreased cell viability and enhanced sensitivity to carmustine treatment. CDKN2A inhibition promoted self-renewal capacity and increased pluripotency marker expression in U87 cells. CDKN2A upregulation led to elevated protein levels of p16INK4a, p14ARF, P53, and P21, which are involved in cell cycle regulation. CDKN2A downregulation in U87 cells was associated with high promoter methylation, which was reversed by treatment with a demethylating agent.

**Conclusion:**

Our findings demonstrate that CDKN2A downregulation in glioblastoma cells is associated with decreased cell viability, enhanced drug resistance, increased self-renewal capacity, and altered expression of pluripotency markers. The observed CDKN2A expression changes are mediated by promoter methylation. These results highlight the potential role of CDKN2A as a therapeutic target and prognostic marker in glioblastoma.

**Supplementary Information:**

The online version contains supplementary material available at 10.1007/s11033-024-09247-5.

## Introduction

Glioblastoma, the most common and aggressive primary brain tumor in adults, poses significant challenges in terms of prognosis and treatment outcomes[1–3]. Despite advances in surgical techniques, radiotherapy, and chemotherapy, the median survival of glioblastoma patients remains dismal, highlighting the urgent need for a deeper understanding of the molecular mechanisms driving tumor development and progression[4]. One gene that has attracted considerable attention in glioblastoma research is CDKN2A, which encodes multiple tumor suppressor proteins involved in cell cycle regulation and senescence[5, 6].

CDKN2A, located on chromosome 9p21, is a complex locus that produces two distinct proteins: p16INK4a (p16) and p14ARF (p14)[7]. These proteins play critical roles in controlling cell proliferation and suppressing tumorigenesis. P16 functions as a cyclin-dependent kinase inhibitor, specifically targeting cyclin-dependent kinase 4 (CDK4) and CDK6, thereby preventing their interaction with cyclin D and inhibiting the phosphorylation of retinoblastoma protein (Rb)[8–10]. This leads to cell cycle arrest at the G1 phase and halts cell proliferation. On the other hand, p14ARF acts as a stabilizer of p53, a master regulator of cell cycle checkpoints and apoptosis. By preventing p53 degradation, p14ARF enhances p53-mediated cell cycle arrest and apoptosis in response to cellular stress[11].

Genetic alterations affecting CDKN2A, such as homozygous deletions, point mutations, or promoter methylation, have been identified in various cancer types, including glioblastoma[12]. Loss of CDKN2A function has been associated with uncontrolled cell proliferation, decreased response to chemotherapy, and poor prognosis in glioblastoma patients[13]. However, the precise mechanisms underlying CDKN2A dysregulation and its functional significance in glioblastoma remain incompletely understood. Recent studies have begun to shed light on the role of CDKN2A in glioblastoma pathogenesis. Downregulation of CDKN2A has been reported in glioblastoma cell lines and patient samples, implicating its tumor-suppressive function[14]. Additionally, experimental studies have demonstrated that restoration of CDKN2A expression or activation of its downstream effectors, such as p53 and p21, can induce cell cycle arrest, inhibit cell proliferation, and promote apoptosis in glioblastoma cells[15, 16]. These findings suggest that targeting CDKN2A and its associated pathways may hold therapeutic potential in glioblastoma treatment.

Furthermore, emerging evidence suggests that CDKN2A may play a role in the maintenance of glioblastoma stem cells (GSCs), a subpopulation of cells with self-renewal capacity and pluripotency that contribute to tumor initiation, growth, and therapy resistance[17]. GSCs are believed to drive tumor recurrence and are associated with poor clinical outcomes[18, 19]. Understanding the regulatory mechanisms governing GSCs and their interaction with CDKN2A could provide crucial insights into glioblastoma progression and identify novel therapeutic strategies.

In this study, we aimed to investigate the functional significance and regulatory mechanisms of CDKN2A in glioblastoma. Specifically, we examined the expression levels of CDKN2A in glioblastoma cells and assessed its impact on cell viability, chemosensitivity, self-renewal capacity, and pluripotency. We also explored the epigenetic regulation of CDKN2A, focusing on promoter methylation as a potential mechanism for its downregulation in glioblastoma. Furthermore, we investigated the effects of CDKN2A modulation on key cell cycle regulators, such as p16INK4a, p14ARF, p53, and p21, to gain mechanistic insights into the tumor-suppressive function of CDKN2A in glioblastoma. The findings from this study contribute to our understanding of the molecular underpinnings of glioblastoma and provide insights into the potential therapeutic targeting of CDKN2A. By elucidating the role of CDKN2A in glioblastoma cell behavior and its interaction with important signaling pathways, we aim to pave the way for the development of novel treatment strategies that could improve patient outcomes in this devastating disease.

## Materials and methods

### Cell culture and treatment

The U87 glioblastoma cell line (ATCC, catalog number HTB-14) was obtained from the American Type Culture Collection (ATCC) and maintained in Dulbecco's Modified Eagle Medium (DMEM) supplemented with 10% fetal bovine serum (FBS, 10,091,148, Gibco™, ThermoFisher Scientific, Waltham, Massachusetts, USA) and 1% penicillin–streptomycin in a humidified incubator at 37 °C with 5% CO2. To investigate the role of CDKN2A in glioblastoma, U87 cells were subjected to CDKN2A inhibition using specific siRNAs or treated with a demethylating agent, 5-aza-2'-deoxycytidine (5-aza-DC) at a concentration of 12 μM[20]. Human astrocytes, specifically normal human astrocytes (NHA), were isolated from the spinal cord and obtained from ScienCell (Cat:#1820, USA). These NHA cells served as a representative model of normal astrocytes and were utilized in this study. The NHA cells were cultured in Astrocyte medium (Cat:#1801, ScienCell) which consisted of a basal medium supplemented with Astrocyte Growth Supplement (ScienCell, Cat:#1852) and 15% fetal calf serum (GBICO, USA). Additionally, the culture medium was supplemented with 100 units/mL penicillin and 100 μg/mL streptomycin to maintain sterility. The cells were cultured in a humidified incubator at 37 °C with 5% CO2 to provide an optimal growth environment.

## CDKN2A inhibition

For CDKN2A knockdown, U87 cells were transfected with CDKN2A-targeting small interfering RNAs (siRNAs) using a transfection reagent Lipofectamine 2000™ (Invitrogen, Cat:#11,668,019) according to the manufacturer's instructions. Non-targeting siRNAs were used as negative controls. The efficiency of CDKN2A knockdown was assessed by quantitative real-time polymerase chain reaction (qRT-PCR) and Western blot analysis.

## CDKN2A overexpression

To investigate CDKN2A overexpression, U87 cells were transfected with plasmids using Lipofectamine 2000™ transfection reagent (Invitrogen, Cat:#11,668,019) according to the manufacturer's instructions. In the Vector group, U87 cells were transfected with the negative control plasmid pcDNA3.1, while in the CDKN2A group, U87 cells were transfected with the pcDNA3.1-CDKN2A plasmid containing the CDKN2A gene. Following transfection, the cells were incubated in the culture medium for an appropriate period to ensure efficient uptake and expression of the plasmid DNA. The efficiency of CDKN2A overexpression was evaluated using qRT-PCR and Western blot analysis.

## Carmustine Treatment

To evaluate the effect of CDKN2A modulation on glioblastoma cell sensitivity to chemotherapy, U87 cells were treated with the chemotherapeutic agent carmustine. Cells were pre-transfected with CDKN2A siRNAs or non-targeting siRNAs or vector plasmid or CDKN2A plasmid for 24 h and then exposed to various concentrations of carmustine (0, 12.5, 25, 50, 100, and 200 μM) for an additional 48 h[21]. Cell viability was determined using MTT assay.

## MTT assay

The MTT assay was performed to assess cell viability. At the end of the specified treatment time point, the culture medium was aspirated from each well. MTT solution (final concentration: 0.5 mg/ml, roche, cat.no.11465007001) was added to each well, and the plates were incubated at 37 °C for 2–4 h to allow the formation of formazan crystals by viable cells. After the incubation period, the MTT solution was carefully removed, and the formazan crystals were dissolved in dimethyl sulfoxide (DMSO). The absorbance of the formazan solution was measured using a microplate reader at a wavelength of 570 nm, with a reference wavelength of 630 nm. The absorbance values obtained from each well were used to calculate the relative cell viability compared to the control group. To calculate the IC50 values for the different treatment groups, the absorbance values obtained from the MTT assay at various carmustine concentrations should be analyzed. The IC50 represents the concentration of carmustine required to inhibit 50% of cell viability[21].

## Sphere-forming assay

To assess the impact of CDKN2A inhibition on the self-renewal capacity of glioblastoma cells, a sphere-forming assay was performed. Sphere-forming assays were conducted following a previously described protocol[22]. In summary, a total of 1.0 × 10^3^ cells were plated in 96-well plates (Corning Inc., Corning, USA) using a serum-free DMEM/F12 medium (Invitrogen) supplemented with 20 ng/mL of basic fibroblast growth factor (bFGF; Peprotech, Rocky Hill, NJ, USA), 1 × Penicillin–Streptomycin Solution, 20 ng/mL of epidermal growth factor (EGF; Peprotech), 10 μg/mL of heparin (Sigma-Aldrich, St. Louis, USA), and 1% B-27 (Life Science). After 1 week, the tumor spheres were assessed in terms of their size and quantity.

## Quantitative real-time polymerase chain reaction (qRT-PCR)

The mRNA expression levels were quantified using qRT-PCR, following a previously reported method[23]. In summary, cellular RNA was extracted using Trizol reagent (Life Technologies, USA) and subsequently reverse transcribed into cDNA using a Takara kit (NHK, Japan). RT-qPCR was performed in a LightCycler 480 instrument (Roche Diagnostics, Switzerland) using SYBR® Premix Ex Taq™ according to the manufacturer’s instructions. GAPDH was used as an internal control. The relative gene expression was calculated using the 2^ΔΔCt^ method. Primer sequences of CDKN2A and GAPDH were as follow: CDKN2A, Forward: 5´-CGACCCTGTCCCTCAAATCC-3´Reverse: 5´-TATCGCGGAGGAAGGAAACG-3´. GAPDH, Forward: 5´-GTGGCTGGCTCAGAAAAAGG-3´, Reverse: 5´-GGGGAGATTCAGTGTGGTGG-3´.

## Western blot analysis

Protein extraction was performed using RIPA lysis buffer, and protein quantification was carried out using a BCA protein assay (Abcam, Cat:# ab102536). The protein samples were then separated on SDS-PAGE gels ranging from 8 to 15% in concentration and subsequently transferred to polyvinylidene fluoride membranes (Immobilon-P, Millipore, Bedford, USA, Cat:# IPVH15150). To block the membranes, a 5% non-fat milk solution in TBST was applied for 1 h at room temperature. The membrane was incubated with primary antibodies against CDKN2A/P14ARF (Abcam, Cat:# ab185620), p16INK4a (Abcam, Cat:# ab241543), P53 (Abcam, Cat:# ab32049), P21(Abcam, Cat:# ab109199), SOX2 (Abcam, Cat:# ab97959), Oct4 (Abcam, Cat:# ab200834), NANOG (Abcam, Cat:# ab109250), KIF4 (Abcam, Cat:# ab122227), or GAPDH (Abcam, Cat:# ab8245) overnight at 4 °C. On the following day, the membranes were washed three times with TBST and the membranes were incubated with horseradish peroxidase-conjugated anti-rabbit or anti-mouse IgG for 1 h at room temperature. Finally, protein visualization was achieved using enhanced chemiluminescence (SuperSignal ECL, ThermoFisher Scientific, USA, Cat:# 32,106) and analyzed using ImageJ software.

## Real-time methylation-specific PCR

DNA extraction was carried out using the PureLinkTM Genomic DNA Mini Kit (ThermoFisher Scientific) following the manufacturer's instructions. Methylation-specific PCR (MSP) was performed as outlined by Herman et al.[24]. A quantity of 500 nanograms of DNA underwent bisulfite conversion of cytosine to thymine using the EpiTect Bisulfite kit (Qiagen). Real-time PCR was conducted on a 7900HT Applied Biosystems instrument with the Syber Green PCR Master Mix serving as the intercalating dye. The reaction mixtures comprised 25 ng of bisulfite-modified template, 200 nmol/l of each primer, and a final volume of 15 μl. The threshold cycle for the methylated CG reaction (CtCG) and the threshold cycle for the unmethylated reaction (CtTG) were determined. The relative levels of methylated DNA (percentage) in each sample were calculated using the formula Cmeth = 100/[1 + 2(CtCG − CtTG)]%[25]. The CpGenome universal methylated and unmethylated DNA (Millipore Sigma) were utilized as positive and negative controls, respectively. The primer sets described by Herman et al.[24] were employed to amplify the methylated and unmethylated CDKN2A genes. MSP primers were as follow[26] Methylated CDKN2A, M-CDKN2A-F: GTTCGTAGGGTTGTAAGAAGAAAAC, M-CDKN2A-R: GCAAACTAACTAACTCACTCCGC, Unmethylated CDKN2A, U-CDKN2A-F: TTTGTAGGGTTGTAAGAAGAAAATGA, U-CDKN2A-R: CACAAACTAACTAACTCACTCCACA.

## 5-aza-2'-deoxycytidine (5-aza-DC) Treatment

To investigate the effect of demethylation on CDKN2A expression and glioblastoma cell behavior, U87 cells were treated with the demethylating agent 5-aza-DC. U87 cells were treated with a concentration of 12 μM 5-Aza-dC for a duration of 96 h. CDKN2A expression was assessed by qRT-PCR and Western blot analysis. Sphere-forming ability and pluripotency marker expression were also evaluated.

## Statistical analysis

Statistical anal- yses were performed using GraphPad Prism version 9.0. All experiments were performed in triplicate, and data are presented as mean ± standard deviation (SD). Statistical analysis was performed using student's t-test or one-way analysis of variance (ANOVA), depending on the experimental design and nature of the data. For comparisons between two groups, Student's t-test was employed. For comparisons among multiple groups, one-way ANOVA was utilized. Subsequently, The Tukey method is used for post hoc multiple comparisons to determine specific differences between group. A p-value of less than 0.05 was considered statistically significant.

## Results

### Low expression of CDKN2A in glioblastoma cells.

**Fig. 1:**
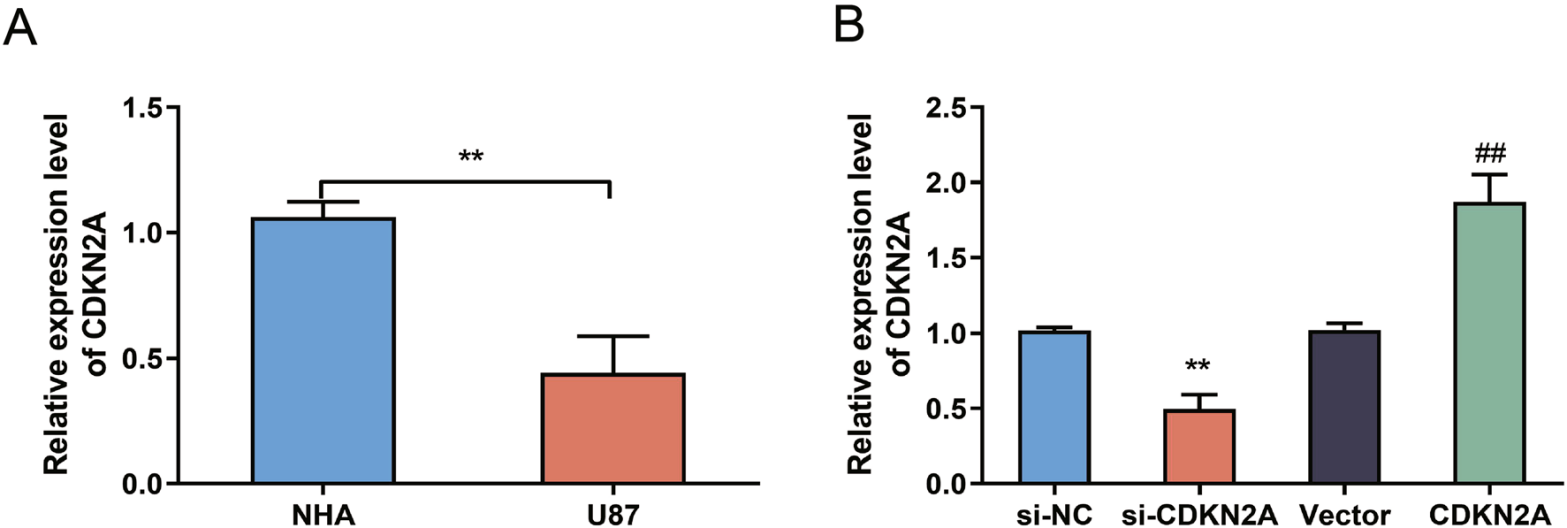
Low expression of CDKN2A in glioblastoma cells. **A** qRT-PCR analysis of CDKN2A expression levels in NHA cells and U87 cells. **B** qRT-PCR analysis of CDKN2A expression levels in si-NC, si-CDKN2A, Vector, and CDKN2A cells.

To investigate the expression levels of CDKN2A in glioblastoma cells, we performed quantitative reverse transcription PCR (qRT-PCR) analysis. Comparing normal human astrocytes (NHA) with U87 glioblastoma cells, we found significantly reduced CDKN2A expression in the U87 cells (Fig. 1A). These findings indicate that CDKN2A is downregulated in glioblastoma cells, suggesting its potential involvement in glioblastoma development and progression.

To further explore the functional significance of CDKN2A downregulation, we conducted additional qRT-PCR experiments in which we manipulated CDKN2A expression levels. Our results revealed significantly decreased CDKN2A expression in the si-CDKN2A group and increased expression in the CDKN2A group compared to si-NC and Vector groups, respectively (Fig. 1B). These observations confirm the successful transfection of CDKN2A into glioblastoma cells, enabling us to investigate its functional implications in subsequent experiments.

## CDKN2A overexpression reduces viability and enhances sensitivity to carmustine in U87 glioblastoma cells.

**Fig. 2:**
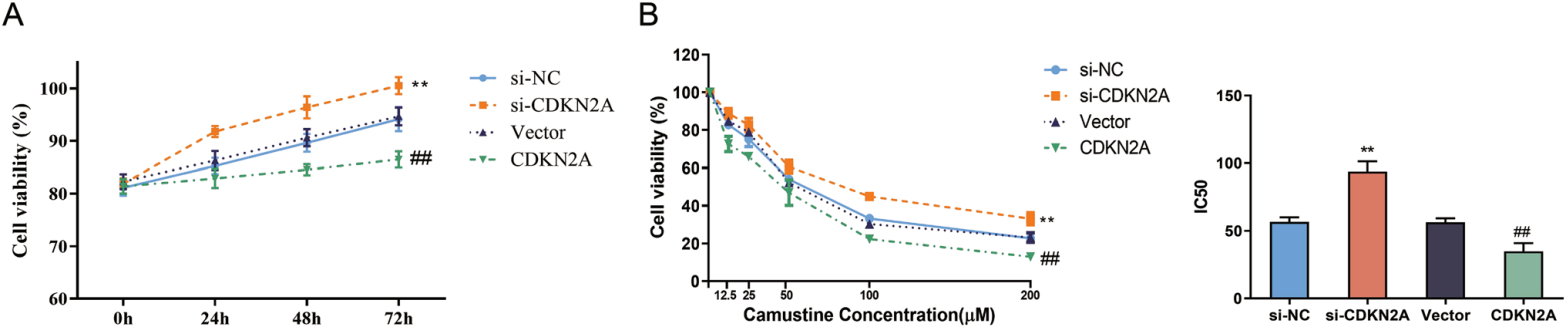
CDKN2A inhibition reduces viability and enhances sensitivity to carmustine in U87 glioblastoma cells. **A** MTT assay measuring cell viability of si-NC, si-CDKN2A, Vector, and CDKN2A cells over time (0, 24, 48, 72h). **B** MTT assay measuring cell viability and IC50 of si-NC, si-CDKN2A, Vector, and CDKN2A cells under different concentrations of carmustine (0, 12.5, 25, 50, 100, 200μM).

To assess the impact of CDKN2A on glioblastoma cell viability, we performed MTT assays at different time points (0, 24, 48, 72 h) following CDKN2A knockdown (si-CDKN2A) or overexpression (CDKN2A). Compared to the Vector and si-NC groups, the CDKN2A group exhibited significantly reduced cell viability over time, indicating a suppressive effect on cell proliferation (Fig. 2A). Conversely, the si-CDKN2A group showed increased cell viability compared to the control groups. These results suggest that CDKN2A overexpression reduces the viability of U87 glioblastoma cells.

Furthermore, we investigated the effect of CDKN2A on the sensitivity of U87 cells to carmustine, a chemotherapeutic agent commonly used in glioblastoma treatment[25]. By subjecting si-NC, si-CDKN2A, Vector, and CDKN2A cells to varying concentrations of carmustine (0, 12.5, 25, 50, 100, 200 μM), we observed that the si-CDKN2A group displayed reduced sensitivity to carmustine compared to the control groups as the concentration of carmustine increased (Fig. 2B). Conversely, the CDKN2A group demonstrated heightened sensitivity to carmustine when compared to the vector groups at escalating carmustine concentrations. Specifically, at a carmustine concentration of 200 μM, we observed a significant decrease in sensitivity to carmustine in the si-CDKN2A group, while the CDKN2A group displayed a substantial increase in sensitivity. These findings are further supported by the observed IC50 values, which were significantly higher in the si-CDKN2A group and significantly lower in the CDKN2A group. These findings suggest that CDKN2A overexpression not only impairs cell viability but also sensitizes U87 glioblastoma cells to carmustine treatment.

## CDKN2A inhibition impairs self-renewal capacity of U87 glioblastoma cells.

**Fig. 3:**
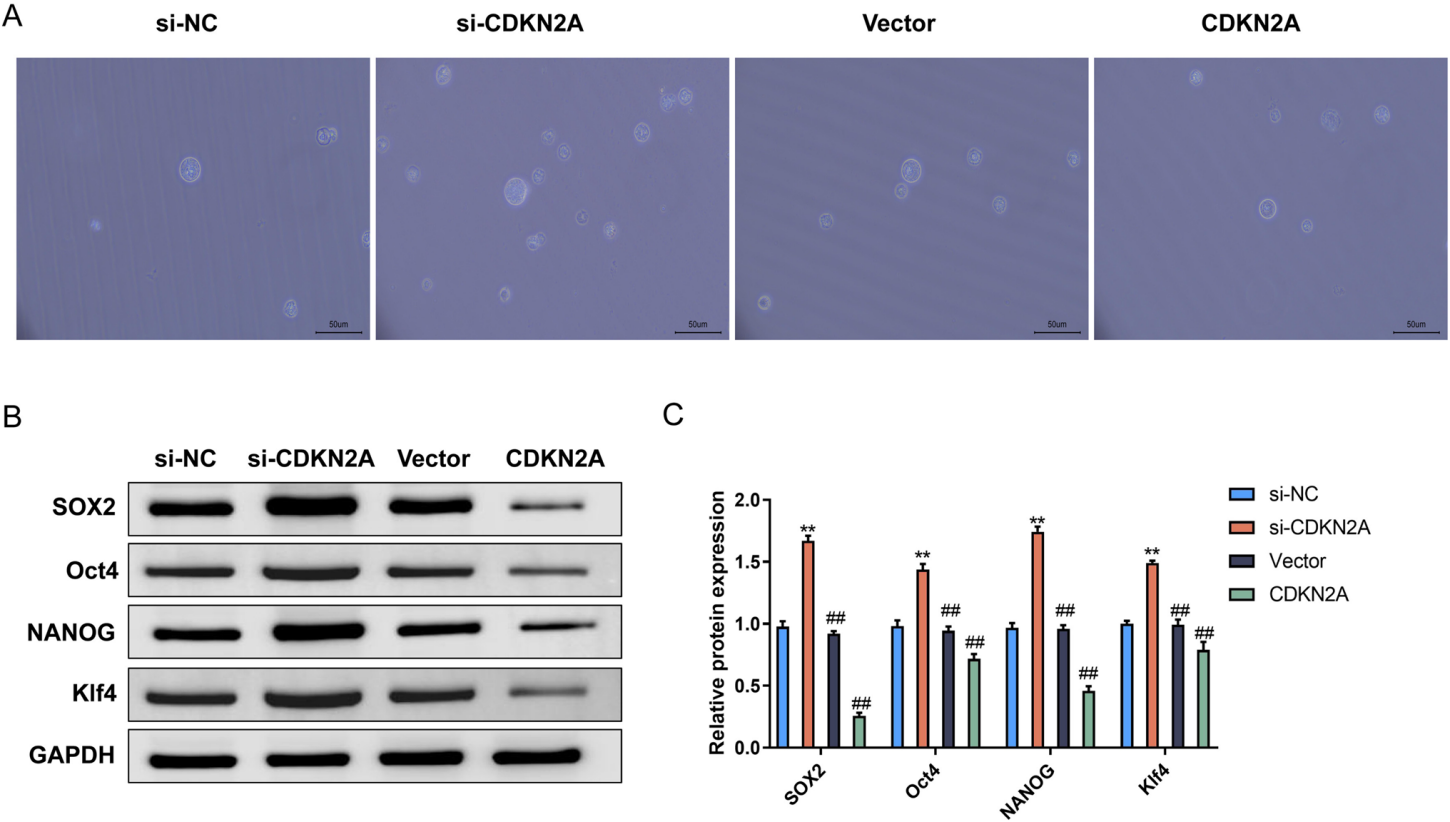
CDKN2A inhibition impairs self-renewal capacity of U87 glioblastoma cells. **A** Sphere formation assay assessing the sphere-forming ability of si-NC, si-CDKN2A, Vector, and CDKN2A cells. **B** Western blot analysis of SOX2, Oct4, NANOG, and KIf4 protein levels in si-NC, si-CDKN2A, Vector, and CDKN2A cells. **C** Analysis of SOX2, Oct4, NANOG, and KIf4 protein levels in the different cell groups.

To investigate the role of CDKN2A in GSC self-renewal, we performed sphere formation assays and assessed the protein levels of key pluripotency markers. In the sphere formation assay, si-CDKN2A, si-NC, Vector, and CDKN2A cells were cultured in non-adherent conditions to promote the formation of tumor spheres. The si-CDKN2A group exhibited significantly enhanced sphere-forming ability compared to the si-NC groups. Conversely, the CDKN2A group showed inhibited sphere-formation of tumor spheres compared to the Vector groups (Fig. 3A). This suggests that CDKN2A overexpression reduces the self-renewal capacity of U87 glioblastoma cells.

We further examined the expression levels of SOX2, Oct4, NANOG, and KIf4, which are critical transcription factors involved in maintaining pluripotency and self-renewal of stem cells. Western blot analysis showed increased levels of SOX2, Oct4, NANOG, and KIf4 proteins in the si-CDKN2A group compared to the si-NC group. In contrast, CDKN2A overexpression significantly reduced protein levels of SOX2, Oct4, NANOG, and KIf4 compared with the Vector group (Fig. 3B and Fig. 3C). These results indicate that CDKN2A inhibition enhances the expression of pluripotency-associated markers in U87 glioblastoma cells, further supporting its role in self-renewal regulation.

## CDKN2A upregulation increases the protein levels of p16INK4a, p14ARF, P53, and P21 in U87 glioblastoma cells.

**Fig. 4:**
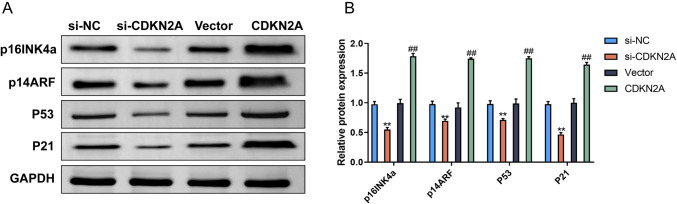
CDKN2A upregulation increases the protein levels of p16INK4a, p14ARF, P53, and P21 in U87 glioblastoma cells. **A** Western blot analysis of p16INK4a, p14ARF, P53, and P21 protein levels in si-NC, si-CDKN2A, Vector, and CDKN2A cells. **B** Analysis of p16INK4a, p14ARF, P53, and P21 protein levels in the different cell groups.

CDKN2A is known to regulate cell cycle progression and induce cell cycle arrest through its downstream effectors, including p16INK4a, p14ARF, P53, and P21. To investigate the impact of CDKN2A on these proteins in U87 glioblastoma cells, we performed western blot analysis. Our results revealed that CDKN2A upregulation led to increased protein levels of p16INK4a, p14ARF, P53, and P21 compared to the vector groups. However, CDKN2A downregulation led to decreased protein levels of p16INK4a, p14ARF, P53, and P21 compared to the si-NC groups (Fig. 4A and Fig. 4B). These findings suggest that CDKN2A plays a crucial role in regulating the expression of cell cycle regulators in U87 glioblastoma cells, leading to cell cycle arrest and potentially inhibiting tumor growth.

## CDKN2A downregulation is due to high promoter methylation in U87 glioblastoma cells.

To investigate the underlying mechanism of CDKN2A downregulation in U87 glioblastoma cells, we analyzed the CpG islands in the CDKN2A gene promoter and performed methylation-specific PCR (MSP) assays.

**Fig. 5:**
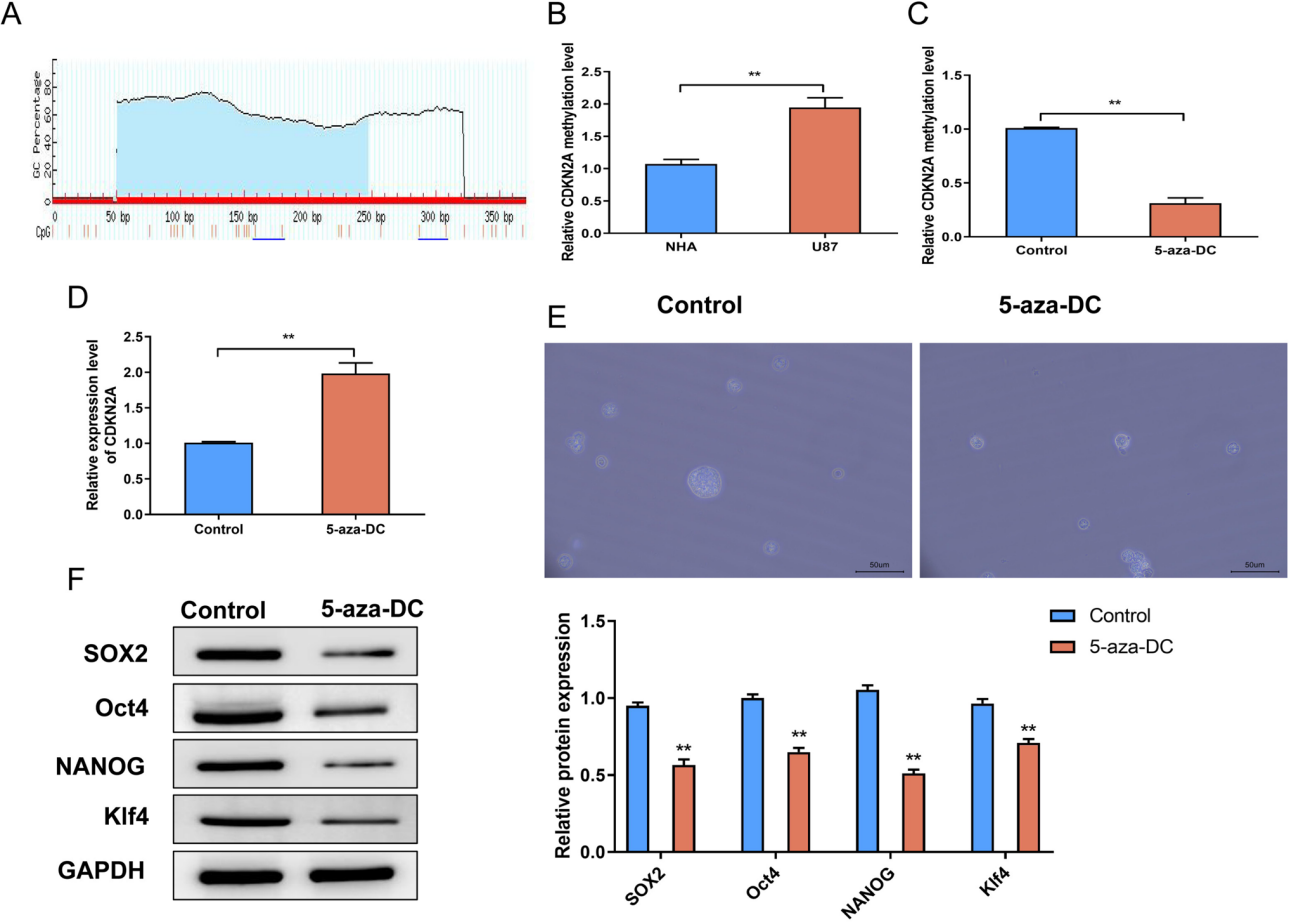
CDKN2A downregulation is due to high promoter methylation in U87 glioblastoma cells. **A** CpG analysis of the CDKN2A gene promoter. **B** Methylation-specific PCR (MSP) analysis of CDKN2A promoter methylation levels in NHA cells and U87 cells. **C** MSP analysis of CDKN2A promoter methylation levels in Control cells and 5-aza-DC cells. **D** qRT-PCR analysis of CDKN2A expression levels in Control cells and 5-aza-DC cells. **E** Sphere formation assay assessing the sphere-forming ability of Control cells and 5-aza-DC cells. **F** Western blot analysis of SOX2, Oct4, NANOG, and KIf4 protein levels in Control cells and 5-aza-DC cells.

The CpG analysis of the CDKN2A gene promoter revealed the presence of multiple CpG sites (Fig. 5A), indicating the potential involvement of DNA methylation in the regulation of CDKN2A expression. MSP analysis showed significantly higher CDKN2A promoter methylation levels in U87 glioblastoma cells compared to normal human astrocytes (NHA) (Fig. 5B). These results suggest that DNA methylation contributes to the downregulation of CDKN2A in U87 glioblastoma cells. To further substantiate the involvement of methylation in the regulation of CDKN2A, we subjected U87 cells to treatment with the demethylating agent 5-aza-2'-deoxycytidine (5-aza-DC) in the experimental groups. In the control groups, U87 cells did not receive any treatment. Subsequently, we conducted MSP assays and qRT-PCR analysis. The 5-aza-DC treatment resulted in reduced CDKN2A promoter methylation levels (Fig. 5C) and increased CDKN2A expression (Fig. 5D) compared to the control group (Control cells), indicating a restoration of CDKN2A expression. Moreover, sphere formation assays revealed that 5-aza-DC treatment significantly inhibited the self-renewal capacity of U87 cells (Fig. 5E). Western blot analysis further demonstrated decreased protein levels of SOX2, Oct4, NANOG, and KIf4 in the 5-aza-DC-treated cells compared to the control group (Fig. 5F). These results suggest that CDKN2A downregulation, mediated by promoter methylation, contributes to the maintenance of self-renewal capacity and pluripotency in U87 glioblastoma cells.

## Discussion

The results presented in this study provide compelling evidence regarding the functional significance and regulatory mechanisms of CDKN2A in glioblastoma. Our findings demonstrate that CDKN2A is significantly downregulated in glioblastoma cells, suggesting its potential role in glioblastoma development and progression. The observed reduction in CDKN2A expression in U87 glioblastoma cells is consistent with previous reports highlighting the tumor-suppressive function of CDKN2A in glioblastoma[27, 28]. For example, Minami et al. [27] conducted studies that revealed how the deletion of CDKN2A reshapes lipid metabolism, making glioblastoma cells more susceptible to ferroptosis. Furthermore, Hsu et al. [28] demonstrated that patients with CDKN2A deletions and NF1 mutations exhibited poorer overall survival rates, whereas patients with CDKN2A wild-type status showed improved overall survival in glioblastoma cases.

Notably, our study elucidates the impact of CDKN2A modulation on glioblastoma cell viability and sensitivity to the chemotherapeutic agent carmustine. CDKN2A inhibition led to a significant increase in cell viability and decreased sensitivity to carmustine treatment in U87 glioblastoma cells. These findings suggest that CDKN2A may play a crucial role in inhibiting cell survival and enhancing sensitivity to chemotherapy in glioblastoma. Carmustine, an established treatment option for glioblastoma, has demonstrated effectiveness in previous studies. In light of this, our research aims to explore strategies for enhancing chemotherapy sensitivity in glioblastoma. Notably, recent investigations have identified novel genes and highlighted the successful application of carmustine in glioblastoma patients. For instance, Radtke et al.[29] have elucidated the impact of ABCB1 loss on the response to carmustine, particularly its lipophilic formulation. Their findings shed light on the potential role of ABCB1 in modulating carmustine efficacy. Additionally, Manini et al.[30] have reported promising results by employing carmustine wafers as adjuvant treatment by inserting them into the resection cavity. Furthermore, Ohnishi T et al.[31] have conducted research suggesting that a high concentration of carmustine could potentially offer survival benefits for less-invasive types of glioblastoma. Their findings indicate the potential of carmustine in improving outcomes for a specific subgroup of patients with less-invasive tumors. Hence, the pursuit of strategies targeting the discovery of novel biomarkers, such as CDKN2A, represents a promising avenue with therapeutic implications. These approaches have the potential to enhance treatment outcomes and augment the sensitivity of carmustine in the context of glioblastoma patients..

Furthermore, our study provides insights into the involvement of CDKN2A in the regulation of self-renewal capacity and pluripotency in glioblastoma cells. CDKN2A inhibition resulted in enhanced sphere-forming ability, a characteristic feature of glioblastoma stem cells (GSCs) that contribute to tumor initiation and recurrence[32]. Additionally, the downregulation of CDKN2A led to increased protein levels of key pluripotency markers, including SOX2, Oct4, NANOG, and KIF4, suggesting a potential role of CDKN2A in maintaining the stemness properties of GSCs[33]. A previous study[34] has additionally documented the significance of stem cell transcription factors, including Oct4, Sox2, Nanog, and KIF4, in the progression of lung cancer. These factors are known to exert a pivotal role in the development and maintenance of stem-like properties within cancer cells, thereby contributing to the intricate dynamics of lung cancer progression. Furthermore, Fatma et al.[34] revealed that the presence of stem cell factors such as OCT4, NANOG, and SOX2, in combination with the activation of diverse signaling pathways, enables cancer cells to sustain a range of phenotypes, thereby fostering both intra- and inter-tumoral heterogeneity. This intricate network of factors and pathways contributes to the dynamic nature of cancer cells, allowing them to adapt and evolve, ultimately shaping the heterogeneous landscape within and among tumors. These findings highlight CDKN2A as a potential regulator of GSCs and suggest that targeting CDKN2A may disrupt the self-renewal capacity and pluripotency of GSCs, thereby impeding tumor growth and recurrence.

Meanwhile, our study demonstrates that CDKN2A upregulation in U87 glioblastoma cells is associated with increased protein levels of p16INK4a, p14ARF, P53, and P21, which are critical cell cycle regulators[35]. CDKN2A is known to induce cell cycle arrest through the activation of these downstream effectors, thereby halting cell proliferation and inhibiting tumor growth[36]. The observed upregulation of these cell cycle regulators provides mechanistic insights into the tumor-suppressive function of CDKN2A in glioblastoma.

Importantly, our study also unravels the epigenetic regulation of CDKN2A in glioblastoma cells. We observed significant elevation in promoter methylation levels within the CDKN2A gene. It is well established that heightened methylation in the promoter region of CDKN2A can exert an inhibitory effect on its expression. Consequently, the downregulation of CDKN2A expression is anticipated in U87 glioblastoma cells as a result of this increased methylation. Treatment with the demethylating agent 5-aza-2'-deoxycytidine (5-aza-DC) effectively reversed CDKN2A silencing, leading to reduced promoter methylation and increased CDKN2A expression. A previous study[37] has provided compelling evidence regarding the involvement of epigenetic modifications, specifically high methylation rates, in the development of recurrent respiratory papillomatosis. Notably, the CDKN2A/TP53 pathway appears to be particularly influenced by this epigenetic modulation. Additionally, Sinha et al.[38] conducted research that revealed the association of frequent deletion and methylation events at the SH3GL2 and CDKN2A loci with both early- and late-onset breast carcinoma. These findings underscore the significance of methylation in the pathogenesis of tumors and highlight the potential role of CDKN2A in their development. Importantly, 5-aza-DC treatment also inhibited the self-renewal capacity of U87 cells and decreased the expression of pluripotency markers, further emphasizing the role of CDKN2A and DNA methylation in maintaining the stemness properties of GSCs.

However, there are several shortcomings that need to be addressed. Firstly, the expression downregulation of CDKN2A observed in glioblastoma cells may not encompass all the functions and regulatory mechanisms of CDKN2A in glioblastoma, as the study focuses primarily on its role in methylation. Other regulatory mechanisms, such as histone modifications, non-coding RNAs, and transcription factor interactions, may also play important roles in the expression regulation of CDKN2A. Therefore, a more comprehensive understanding of the complete regulatory mechanisms of CDKN2A in glioblastoma is warranted. Second, the study heavily relies on cell lines and in vitro experiments, without validation in animal models or clinical samples. Further research is needed to determine the exact role and potential applications of CDKN2A in glioblastoma development and treatment. Third, although the study provides important insights into various aspects of CDKN2A in glioblastoma, there are still unanswered questions. For instance, the precise mechanisms of CDKN2A in glioblastoma development and progression remain unclear, as well as how these findings can be translated into therapeutic strategies. In addition, we only conducted this study in a single GBM cell line and did not delve into the complex molecular pathways and downstream effectors by which CDKN2A exerts its tumor suppressor function in glioblastoma. Finally, as temozolomide serves as the gold standard for first-line treatment of GBMs, it is necessary to use temozolomide as a positive drug to clarify the efficacy of carmustine in the treatment of GBMs. Therefore, in the future, it is necessary to validate in multiple GBM cell lines, conduct experiments to deeply explore the mechanisms involved, and simultaneously explore the therapeutic effects of carmustine and temozolomide in in vitro and in vivo experiments.

## Conclusion

Overall, our study provides comprehensive insights into the functional significance and regulatory mechanisms of CDKN2A in glioblastoma. The downregulation of CDKN2A in glioblastoma cells, possibly mediated by promoter methylation, contributes to increased cell viability, chemoresistance, enhanced self-renewal capacity, and maintenance of pluripotency. Conversely, CDKN2A upregulation induces cell cycle arrest and inhibits tumor growth. These findings highlight CDKN2A as a potential therapeutic target for glioblastoma, as strategies aimed at restoring CDKN2A expression or inhibiting its silencing mechanisms may offer promising avenues for the development of novel treatment approaches.

## Supplementary Information

Below is the link to the electronic supplementary material.

Supplementary file1 (PDF 1375 KB)

## Data Availability

The datasets used and/or analysed during the current study available from the corresponding author on reasonable request.
