# Supplementary material for: CDKN2A promoter methylation enhances self-renewal of glioblastoma stem cells and confers resistance to carmustine
Source: Mol Biol Rep. 2024 Mar 5;51(1):385. doi: 10.1007/s11033-024-09247-5 (PMC10912136; doi:10.1007/s11033-024-09247-5)
Supplement: Supplementary file 1 — Supplementary file1 (PDF 1375 KB) [file 11033_2024_9247_MOESM1_ESM.pdf]

Figure 3B

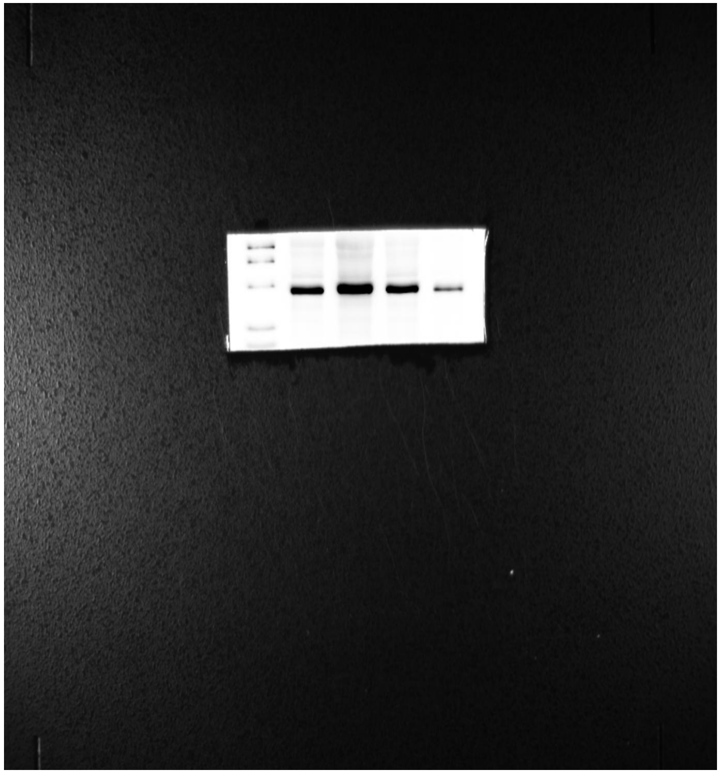

The original Western Blot images of SOX2 in Figure 3B. From left to right: si-NC, si-CDKN2A, Vector, CDKN2A.

Figure 3B

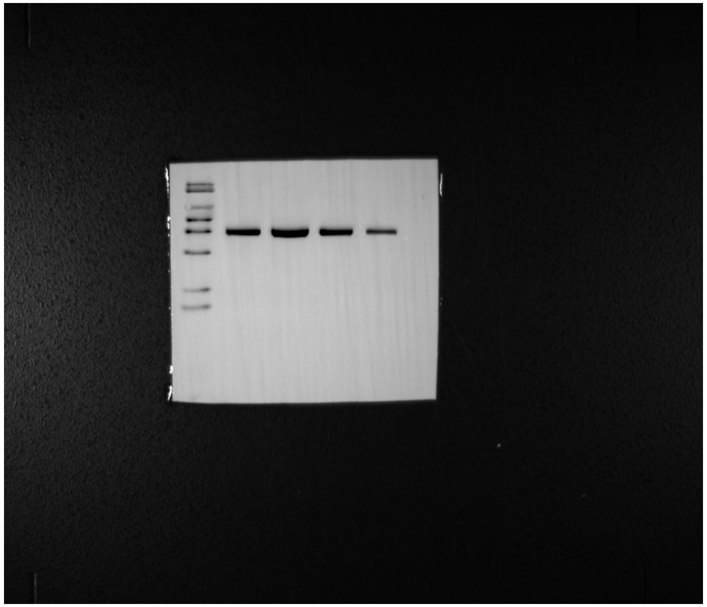

The original Western Blot images of Oct4 in Figure 3B. From left to right: si-NC, si-CDKN2A, Vector, CDKN2A.

Figure 3B

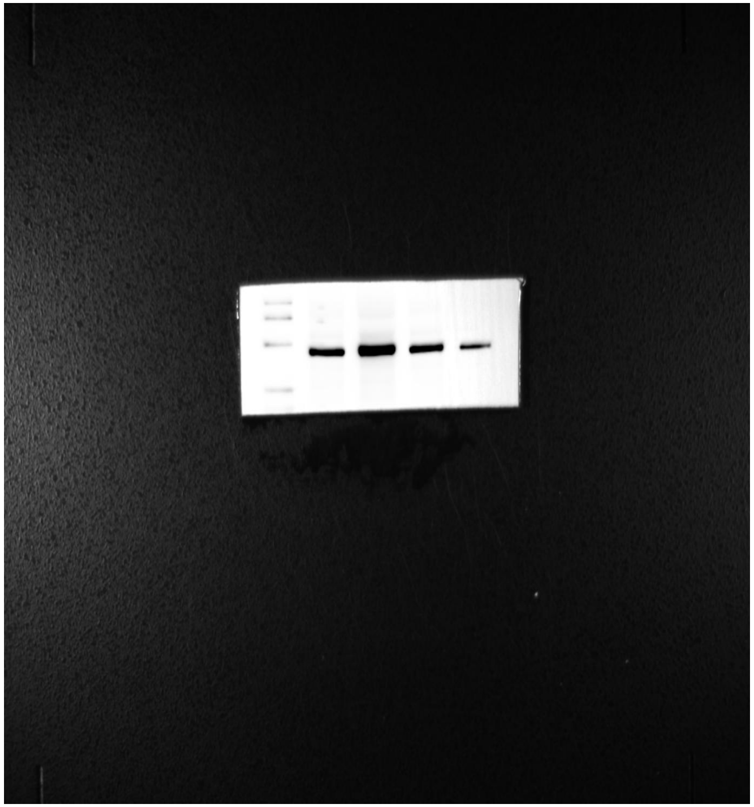

The original Western Blot images of NANOG in Figure 3B. From left to right: si-NC, si-CDKN2A, Vector, CDKN2A.

Figure 3B

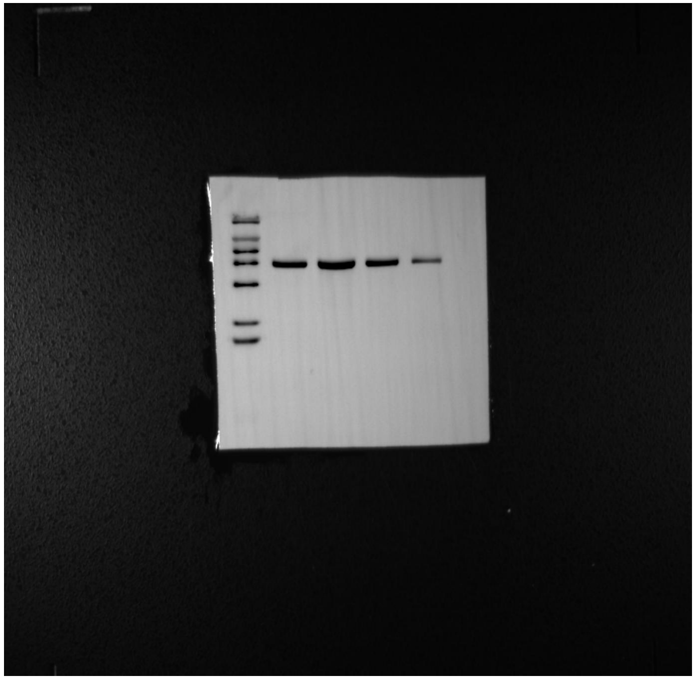

The original Western Blot images of Klf4 in Figure 3B. From left to right: si-NC, si-CDKN2A, Vector, CDKN2A.

Figure 3B

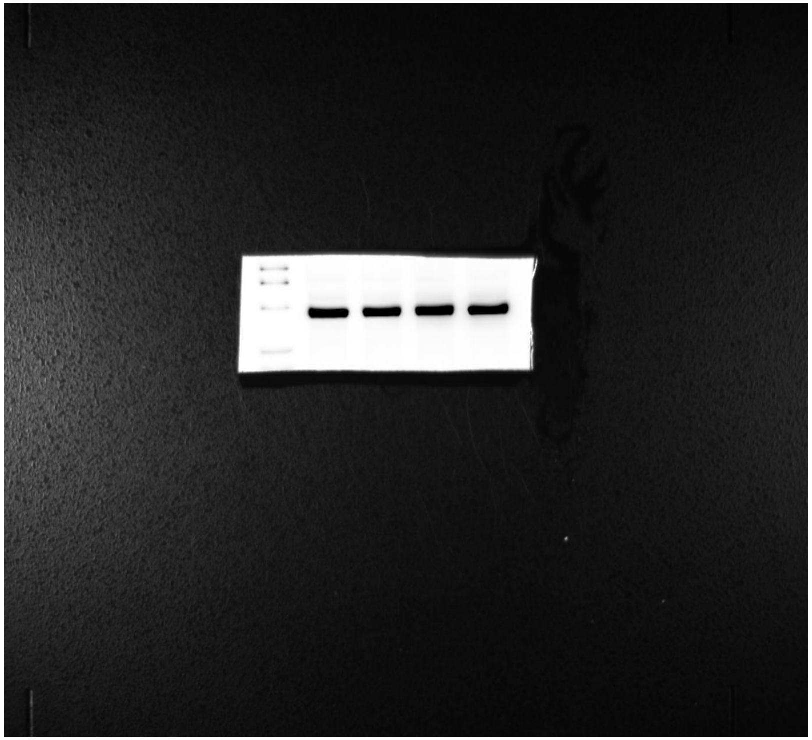

The original Western Blot images of GAPDH in Figure 3B. From left to right: si-NC, si-CDKN2A, Vector, CDKN2A.

Figure 4A

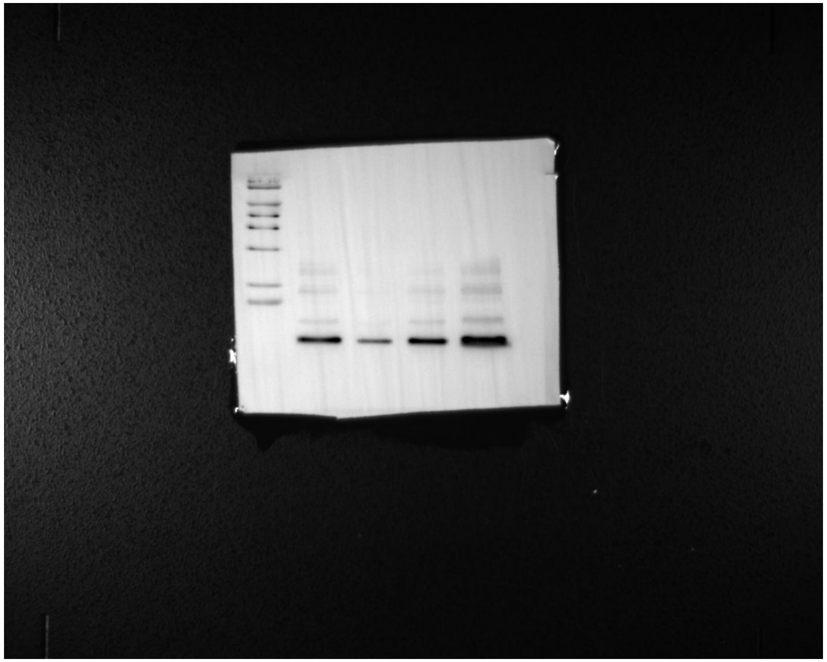

The original Western Blot images of p16INK4a in Figure 4A. From left to right: si-NC, si-CDKN2A, Vector, CDKN2A.

Figure 4A

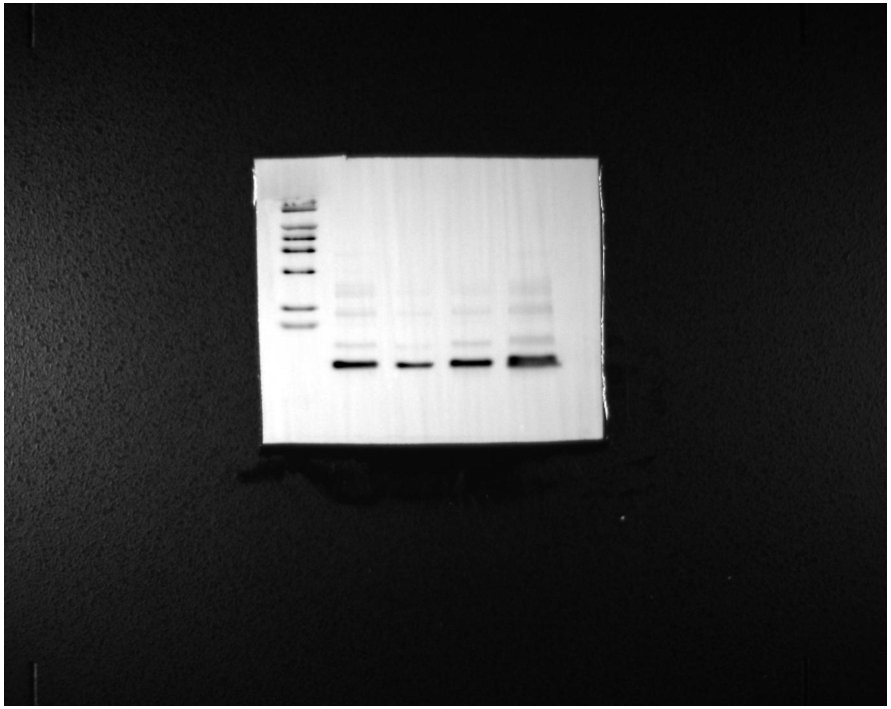

The original Western Blot images of p14ARF in Figure 4A. From left to right: si-NC, si-CDKN2A, Vector, CDKN2A.

Figure 4A

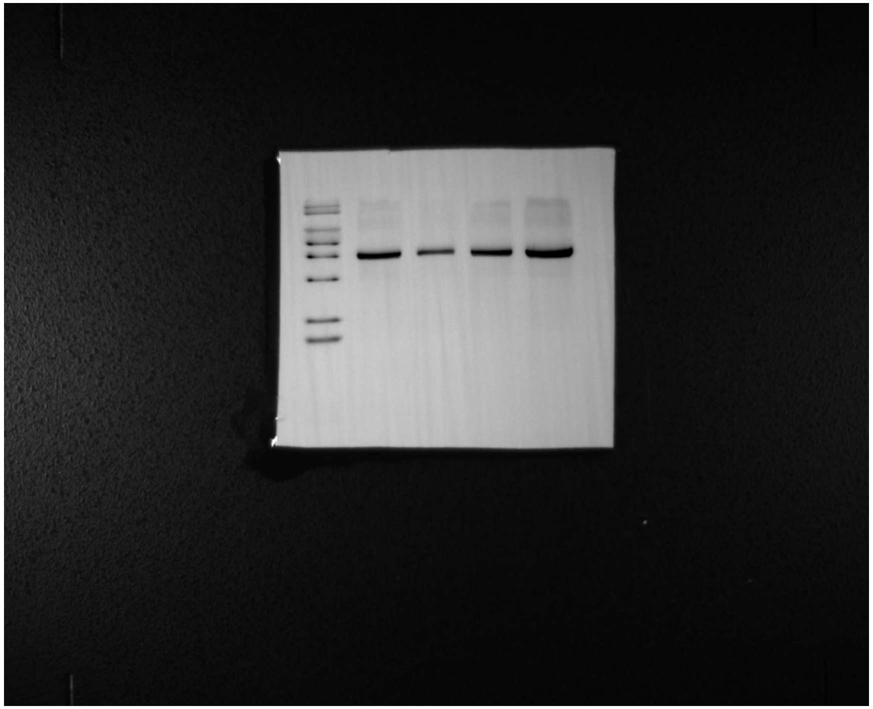

The original Western Blot images of P53 in Figure 4A. From left to right: si-NC, si-CDKN2A, Vector, CDKN2A.

Figure 4A

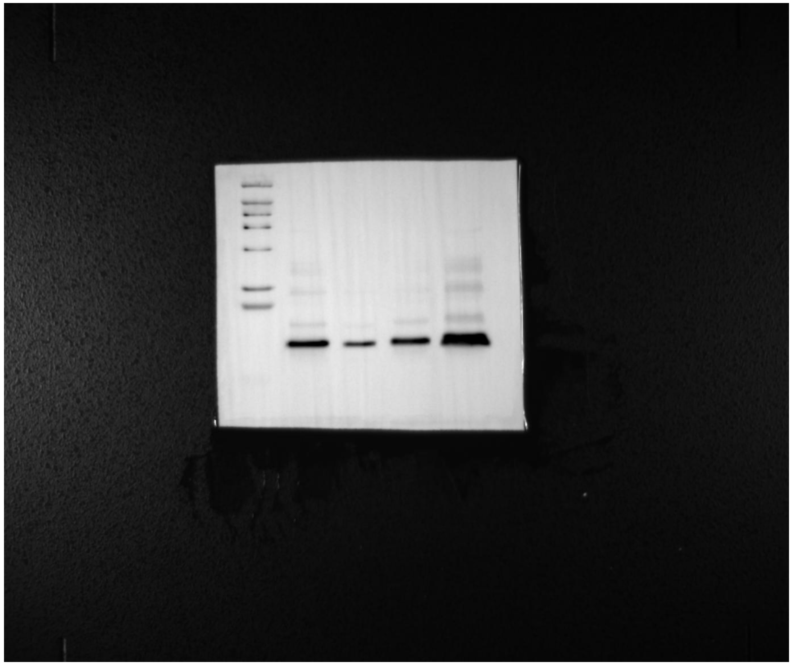

The original Western Blot images of P21 in Figure 4A. From left to right: si-NC, si-CDKN2A, Vector, CDKN2A.

Figure 4A

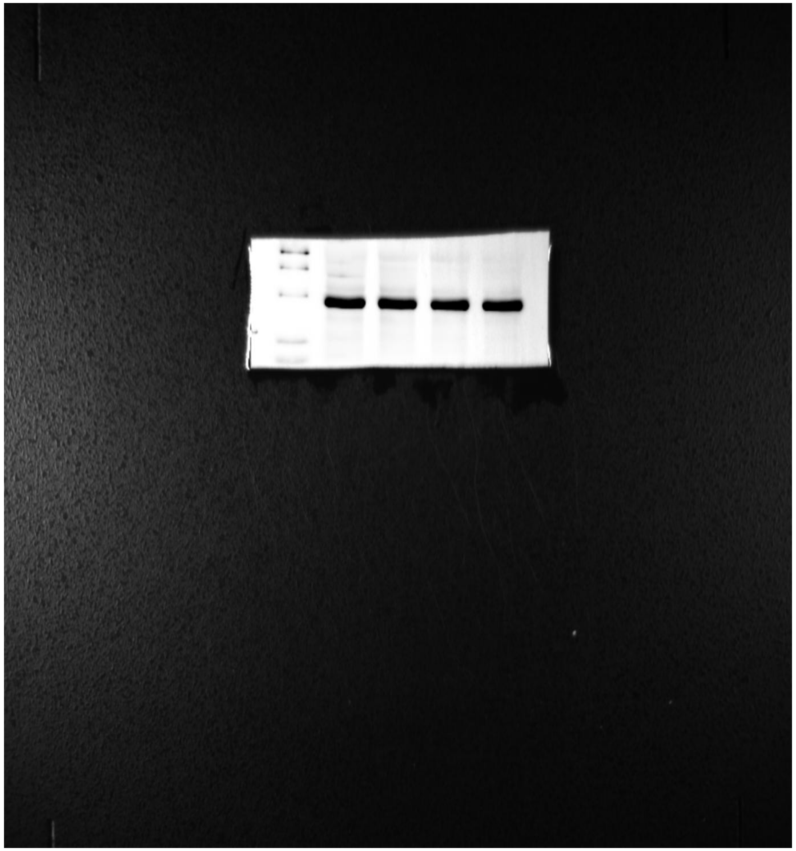

The original Western Blot images of GAPDH in Figure 4A. From left to right: si-NC, si-CDKN2A, Vector, CDKN2A.

Figure 5F

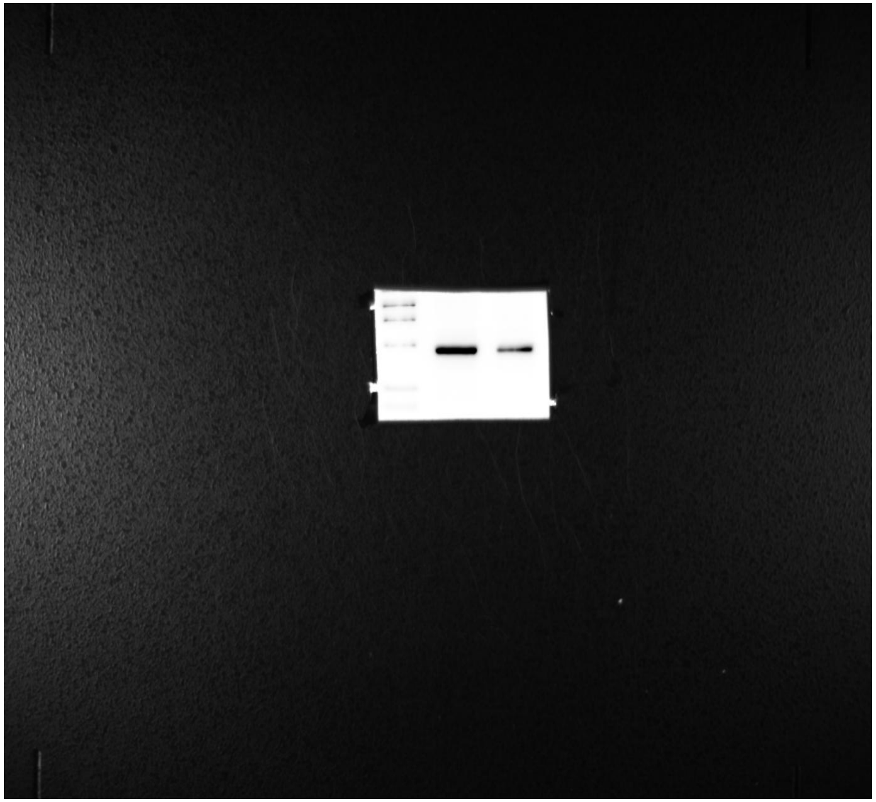

The original Western Blot images of SOX2 in Figure 5F. From left to right: Control, 5-aza-DC.

Figure 5F

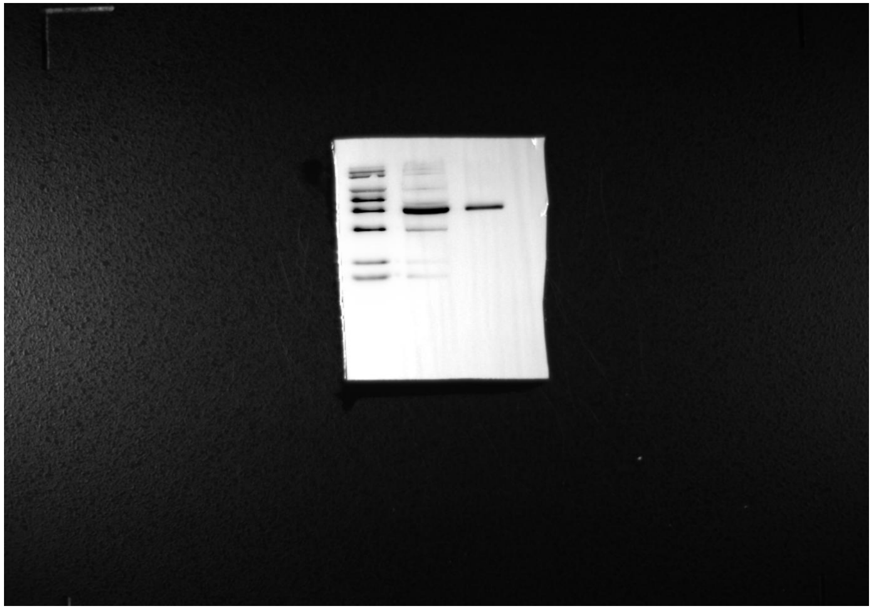

The original Western Blot images of Oct4 in Figure 5F. From left to right: Control, 5-aza-DC.

Figure 5F

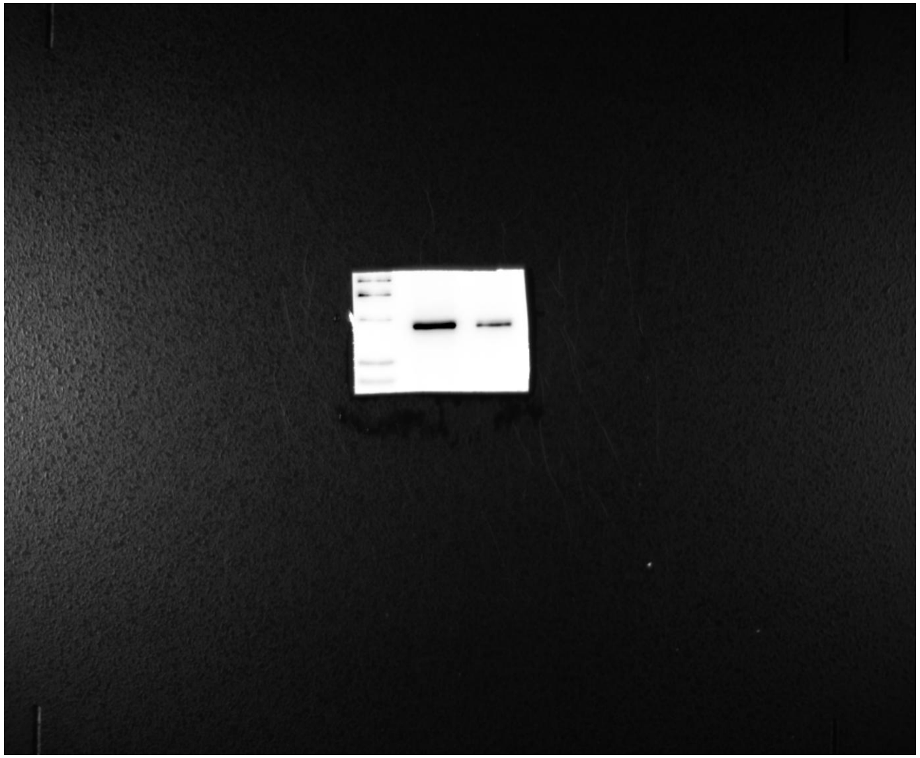

The original Western Blot images of NANOG in Figure 5F. From left to right: Control, 5-aza-DC.

Figure 5F

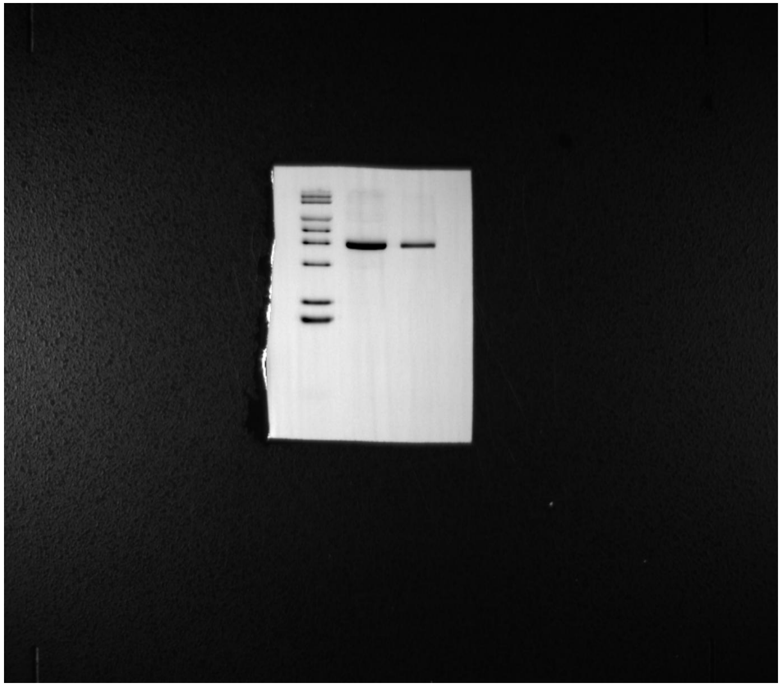

The original Western Blot images of Klf4 in Figure 5F. From left to right: Control, 5-aza-DC.

Figure 5F

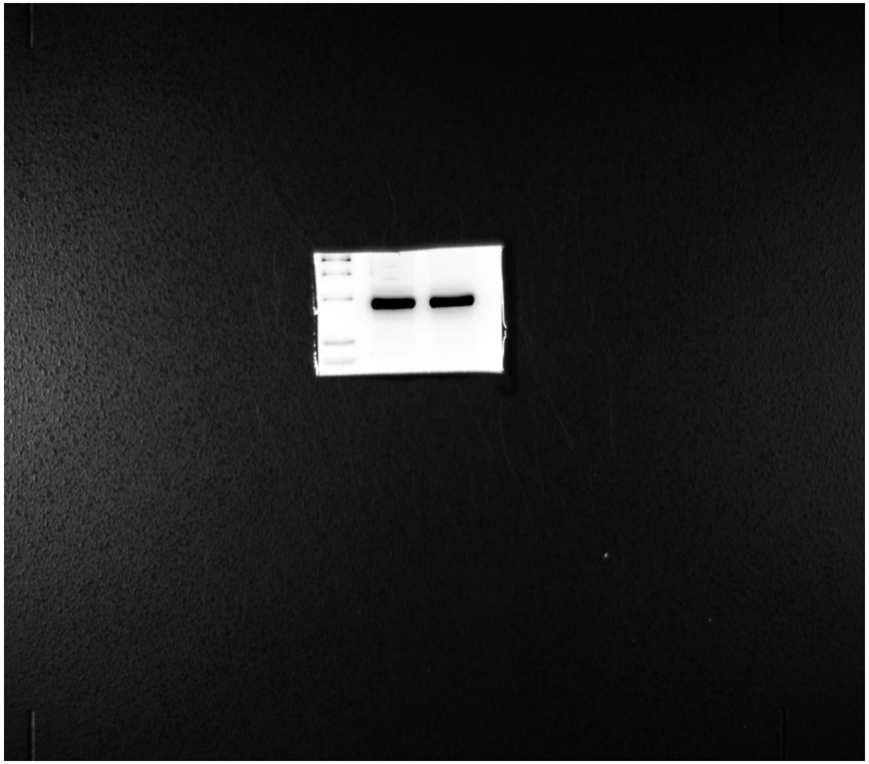

The original Western Blot images of GAPDH in Figure 5F. From left to right: Control, 5-aza-DC.
